# Compartmentalized effects of aging on group 2 innate lymphoid cell development and function

**DOI:** 10.1111/acel.13019

**Published:** 2019-08-20

**Authors:** Shanti S. D'Souza, Xiaofei Shen, Ivan T. H. Fung, Longyun Ye, Marcy Kuentzel, Sridar V. Chittur, Yoichi Furuya, Christian W. Siebel, Ivan P. Maillard, Dennis W. Metzger, Qi Yang

**Affiliations:** ^1^ Department of Immunology and Microbial Disease Albany Medical College Albany New York USA; ^2^ Center for Functional Genomics University at Albany‐SUNY Rensselaer New York USA; ^3^ Department of Discovery Oncology Genentech South San Francisco California USA; ^4^ Division of Hematology‐Oncology, Department of Medicine & Abramson Family Cancer Research Institute University of Pennsylvania Perelman School of Medicine Philadelphia Pennsylvania USA

**Keywords:** aging, influenza, innate lymphoid cells

## Abstract

The effects of aging on innate immunity and the resulting impacts on immunosenescence remain poorly understood. Here, we report that aging induces compartmentalized changes to the development and function of group 2 innate lymphoid cells (ILC2), an ILC subset implicated in pulmonary homeostasis and tissue repair. Aging enhances bone marrow early ILC2 development through Notch signaling, but the newly generated circulating ILC2 are unable to settle in the lungs to replenish the concomitantly declining mature lung ILC2 pool in aged mice. Aged lung ILC2 are transcriptomically heterogeneous and functionally compromised, failing to produce cytokines at homeostasis and during influenza infection. They have reduced expression of *Cyp2e1*, a cytochrome P450 oxidase required for optimal ILC2 function. Transfer of lung ILC2 from young mice enhances resistance to influenza infection in old mice. These data highlight compartmentalized effects of aging on ILC and indicate that targeting tissue‐resident ILCs might unlock therapies to enhance resistance to infections and diseases in the elderly.

## 1 INTRODUCTION

Aging is a complex process in which an organism progressively loses physiologic function leading to increased vulnerability to diseases and death. Aging is associated with various metabolic, genomic, proteostatic, and intercellular changes in many different organs and systems (Lopez‐Otin, Blasco, Partridge, Serrano, & Kroemer, 2013). A prominent manifestation of aging is enhanced susceptibility to infectious diseases and the underlying deterioration of the immune system, termed immunosenescence (Goronzy & Weyand, 2013; Nikolich‐Zugich, 2018). Mechanisms of immunosenescence remain unclear. Aging‐induced changes to adaptive immune cells and the resulting impacts on immunosenescence are well recognized (Goronzy & Weyand, 2013; Nikolich‐Zugich, 2018). However, the effects of aging on the development and function of innate immune cells remain largely unknown.

Aging is associated with diverse and intricate physiologic changes in multiple organs and cellular systems. Lymphoid tissues, such as bone marrow (BM), thymus, and lymph nodes, are highly susceptible to the impacts of aging (Chinn, Blackburn, Manley, & Sempowski, 2012). Aging‐induced BM stromal cell failure and thymus involution result in diminished production of naïve T and B lymphocytes (Chinn et al., 2012). The maintenance and maturation of adaptive lymphocytes are further impaired by age‐related structural and functional changes to the lymph nodes, leading to increased vulnerability to pathogens and poor responses to vaccination (Thompson, Smithey, Surh, & Nikolich‐Zugich, 2017; Turner & Mabbott, 2017). Aging is also associated with changes to nonlymphoid tissues such as the lungs (Lowery, Brubaker, Kuhlmann, & Kovacs, 2013); however, how aging influences tissue immunity remains very poorly understood.

Innate lymphoid cells (ILCs) are a unique family of innate lymphocytes that lack clonally distributed antigen receptors but functionally and molecularly resemble T cells (Vivier et al., 2018). GATA3‐expressing group 2 innate lymphoid cells (ILC2) are the predominant ILC subset in the lung. Lung‐resident ILC2 are implicated in pulmonary homeostasis, epithelial repair, and lung remodeling (Vivier et al., 2018). ILC2 are seeded into the lungs during prenatal or perinatal stages (Gasteiger, Fan, Dikiy, Lee, & Rudensky, 2015; Nussbaum et al., 2013). Mature lung ILC2 in adult young mice are noncirculating tissue‐resident cells, and their local proliferation, but not recruitment from the BM, is a signature of activation (Gasteiger et al., 2015). Nevertheless, BM lLC lymphopoiesis remains active in adult mice (Yang & Bhandoola, 2016). Various ILC and ILC2 precursors have been identified in the BM of adult mice (Yang & Bhandoola, 2016). When transferred to irradiated recipients, these BM ILC/ILC2 precursors efficiently give rise to mature ILC2 in the lungs of recipient mice (Yang & Bhandoola, 2016). However, it remains unknown whether BM precursors may home into the lungs to replenish mature lung ILC2 in physiologic and pathologic conditions other than irradiation. Very little is known about whether and how aging may impact ILC development or function.

In this study, we have explored the effects of aging on ILC2 development and function. Interestingly, our data reveal highly compartmentalized effects of aging on ILC2. Specifically, aging is paradoxically associated with increased early ILC2 development in the BM, but reduced maintenance and function of mature ILC2 in the lungs. Aging leads to a drastic increase in the numbers of BM ILC2 precursors (ILC2P) through Notch signaling‐dependent mechanisms. However, this increase is not sufficient to replenish the concomitant decline in mature ILC2 in the lungs of aged mice. Mature lung ILC2 in aged mice are numerically and functionally compromised, failing to produce cytokines both at homeostasis and during influenza infection. Aged lung ILC2 have reduced fatty acid uptake and decreased expression of peroxisomal and cytochrome p450 (CYP) enzymes that are required for optimal ILC2 function. Transfer of activated ILC2 purified from the lungs of young mice enhances resistance to influenza infection in old mice. Together, these data highlight highly tissue‐specific effects of aging on innate lymphoid cell development and function, and indicate that targeting tissue‐resident ILC might unlock new therapies to enhance resistance to infectious diseases in the elderly.

## 2 RESULTS

### 2.1 Aging enhances early ILC2 development in the bone marrow

**Figure 1:**
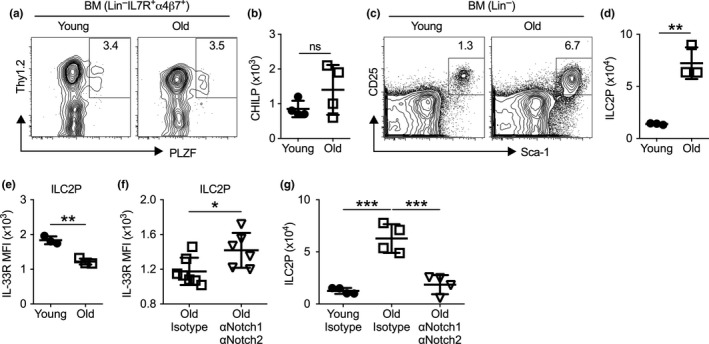
Aging enhances early ILC2 development in the bone marrow. (a) Representative flow cytometry profile of common helper innate lymphoid cells (CHILP, Lin^‒^IL7R^+^α4β7^+^Thy1^+^PLZF^+^) in the bone marrow of young (2–3 months) and old (19–24 months) mice. Plots were pregated on Lin^‒^IL7R^+^α4β7^+^ cells. (b) Number of CHILP from bone marrow of young and old mice. (c) Representative flow cytometry profile of ILC2P (Lin^‒^CD25^+^Sca‐1^+^) from the bone marrow of young and old mice. Plots were pregated on Lin^‒^ cells. (d) Number of ILC2P cell from bone marrow of young and old mice. (e) Mean fluorescence intensity (MFI) of IL‐33R of ILC2P cells. (f) MFI of IL‐33R from old mice treated with isotype controls or αNotch1 and αNotch2 antibodies. (g) Number of ILC2P from young and old mice treated with isotype controls or αNotch1 and αNotch2 for 3 days. Data are from 3 to 6 mice per group; *, *p* < 0.05; **, *p* < 0.01; ***, *p* < 0.001

A recognized mechanism of immunosenescence is diminished adaptive lymphopoiesis leading to declined production of naïve T/B cells (Nikolich‐Zugich, 2018). However, it remains unclear whether such a decline also occurs in early innate lymphoid cell development. Thus, we compared known ILC progenitor numbers in the BM of young (2–3 months) versus old (19–24 months) mice. The average number of PLZF^+^ common helper innate lymphoid progenitors (CHILP) (Constantinides, McDonald, Verhoef, & Bendelac, 2014; Klose et al., 2014) showed a trend toward increasing with age, although the increase was not statistically significant due to heterogeneity in the aged population (Figure 1a,b). In contrast, the number of BM ILC2P (Hoyler et al., 2012) was increased more than fivefold in old mice, suggesting enhanced differentiation toward the ILC2 lineage (Figure 1c,d). The expression of proliferation marker Ki67 was comparable between young and aged BM ILC2P, suggesting that other mechanisms might contribute to the accumulation of ILC2P with aging (Figure S1a). Together, in contrast to declining adaptive lymphopoiesis with aging, aging is associated with increased ILC2 development in the BM.

As a clue to ascertain the signals through which aging increases ILC2 numbers in the BM, we noted that aging correlated with reduced IL‐33R expression in BM ILC2P (Figure 1e). Because Notch signaling can repress IL‐33R expression in ILC2 (Zhang et al., 2017) and may also promote early ILC2 development (Yang et al., 2015, 2013), we hypothesized that aged BM precursors might be driven to the ILC2 lineage through enhanced Notch signaling. Using receptor‐specific blocking antibodies, we confirmed that inhibition of Notch1 and Notch2 partially restored IL‐33R expression in old BM ILC2P (Figure 1f). More importantly, we discovered that antibody treatment also reversed the aging‐induced accumulation of BM ILC2P quickly within three days of treatment (Figure 1g). This rapid decrease in BM ILC2P numbers in aged mice demonstrates that maintaining the higher numbers of BM ILC2 that are observed with aging requires a tonic Notch signal (Figure 1g). Of note, inhibition of Notch1 and Notch2 did not affect BM ILC2P numbers in young mice, suggesting that Notch signaling specifically promotes aging‐associated accumulation of BM ILC2P in old mice (Figure S1b).

### 2.2 Mature ILC2 in the aged lungs are numerically and functionally compromised

**Figure 2:**
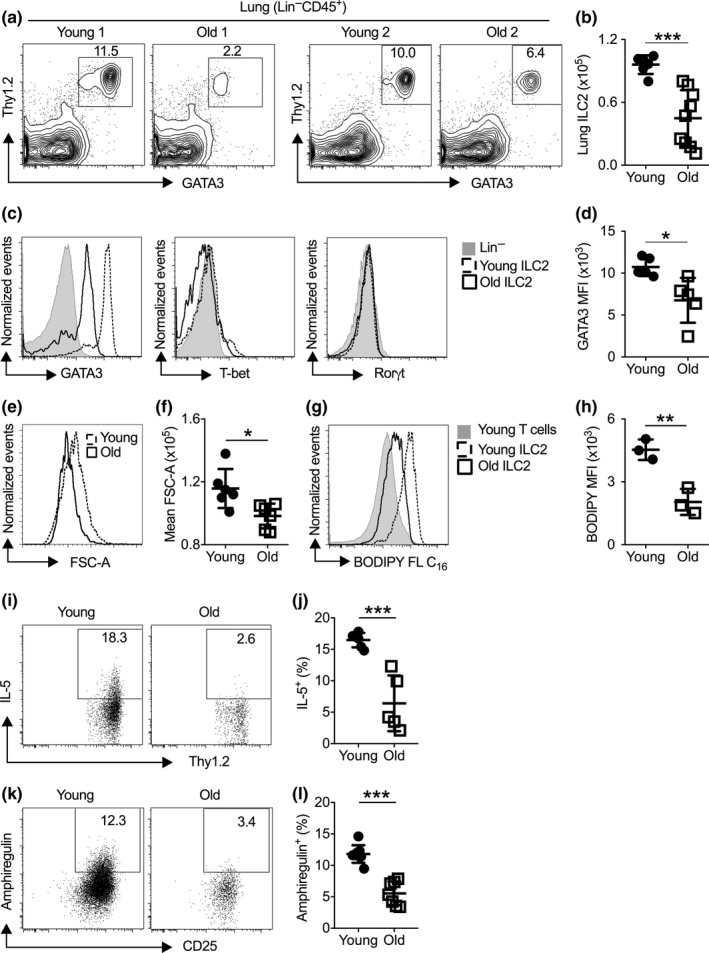
Lung‐resident ILC2 are numerically and functionally compromised with aging. (a) Representative flow cytometry plot of mature ILC2 (Lin^‒^CD45^+^Thy1^+^GATA3^+^) in the lungs of young and old mice. (b) Number of ILC2 in the lungs of young and old mice. (c) Representative histogram of lung ILC2 showing expression of GATA3, T‐bet, or Rorγt. (d) Mean fluorescence intensity (MFI) of GATA3 in lung ILC2 from young and old mice. (e) Representative histogram of FSC‐A of lung ILC2 from young and old mice. (f) Mean FSC‐A of young and aged lung ILC2. (g) Mice were injected with BODIPY FL C_16_, and uptake was measured in ILC2 and T cells from the lungs of young and old mice. (h) Quantification of BODIPY FL C_16_ in T and ILC2 from the lungs of young and old mice. (i) Representative flow cytometry plot showing IL‐5 expression of lung ILC2 in young and old mice. (j) Percentage of IL‐5^+^ ILC2 from lungs of young and old mice. (k) Representative flow cytometry plot showing amphiregulin expression of lung ILC2 in young and old mice. (l) Percentage of amphiregulin^+^ ILC2 from lungs of young and old mice. Data are from 3 to 9 mice per group; *, *p* < 0.05; **, *p* < 0.01; ***, *p* < 0.001

We then examined whether the enhanced BM ILC2 development might lead to increased cellularity of mature ILC2 in the lungs of old mice. We compared the number of mature ILC2, defined by Lin^‐^GATA3^+^Thy1^+^CD45^+^ cells, in the lungs of young and old mice. To our surprise, we did not observe an increase in the numbers of lung‐resident ILC2 with aging. In contrast, the average numbers of mature ILC2 in the lungs were reduced with aging (Figure 2a,b). In addition, remarkable cellular heterogeneity was noted among individual old mice (Figure 2a,b). Some old mice have moderate reduction in the cellularity of lung‐resident ILC2, whereas others were nearly depleted of mature ILC2 in the lung (Figure 2a,b). Together, aging is paradoxically associated with increased early ILC2 development in the BM but reduced maintenance of mature ILC2 in the lung.

We next compared the molecular and functional characteristics of mature ILC2 in the lungs of young and old mice. Lung ILC2 in aged mice expressed GATA3, but at a lower level than young lung ILC2 (Figure 2c,d). Like young ILC2, aged lung ILC2 lacked the expression of molecules characteristic of other ILC lineages, such as T‐bet and Rorγt, indicating that they were committed to the ILC2 lineage (Figure 2c). Both aged and young lung ILC2 expressed KLRG1, verifying that they are mature ILC2 (Figure S2a). Intravenous labeling of anti‐CD45.2 antibody indicated that lung ILC2 from both young and aged mice are noncirculating, tissue‐resident cells (Figure S2b). Previous work has established that mature ILC2 utilize fatty acid metabolism for maintenance and function (Wilhelm et al., 2016). Interestingly, the cell size of aged lung ILC2 was smaller than those of young lung ILC2 (Figure 2e,f), and their fatty acid uptake was markedly reduced (Figure 2g,h), indicating reduced cellular metabolism. Neither young nor aged lung ILC2 took up significant amounts of glucose, arguing against a switch from fatty acid metabolism to glucose metabolism (Figure S2c). Previous studies indicate that mature lung ILC2 in healthy young mice constantly produce IL‐5 and amphiregulin that restrict airway inflammation and protect epithelial barrier integrity (Califano et al., 2018; Guo et al., 2018; Monticelli et al., 2011). We detected homeostatic production of IL‐5 and amphiregulin by lung ILC2 in young mice using intracellular staining, without PMA/ionomycin re‐stimulation (Figure 2i–l). Such homeostatic cytokine production was abolished in aged lung ILC2, indicating a loss of physiologic function of lung ILC2 with aging (Figure 2i–l). Production of IL‐13, another known ILC2 product, was undetectable in either young or aged lung ILC2 (not shown). Together, aging leads to reduced cellularity and functional activity of mature lung ILC2.

### 2.3 BM ILC2P are unable to replenish mature ILC2 in the lungs of old mice

**Figure 3:**
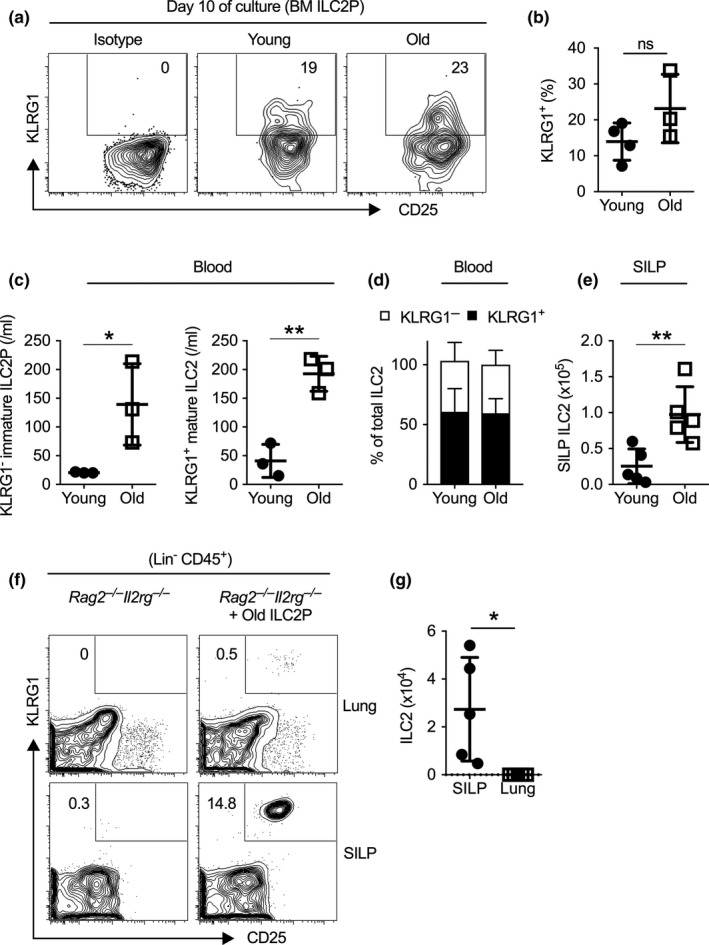
Bone marrow (BM) and circulating ILC2 maturation do not decline with aging. (a) Sort‐purified BM ILC2P were co‐cultured with OP9 stroma in the presence of 10 ng/ml IL‐2, IL‐7, SCF, and IL‐33. Shown are representative flow cytometry plots of KLRG1^+^ mature ILC2 that emerged at day 10 after culture. (b) Percentage of KLRG1^+^ ILC2 that appeared was examined at day 10 after OP9‐co‐culture. (c) Number of immature ILC2 precursor (Lin^‒^Thy1^+^T1/ST2^+^KLRG1^‒^) and mature ILC2 (Lin^‒^Thy1^+^T1/ST2^+^KLRG1^+^) in the peripheral blood of young and old mice. (d) Percentage of KLRG1^+^ and KLRG1^‒^ cells among total ILC2 in the peripheral blood of young and old mice. (e) Number of ILC2 in the small intestinal lamina propria (SILP) of young and old mice. (f) ILC2P from old mice were injected intravenously into *Rag2* ^‒/‒^ *Il2rg* ^‒/‒^ mice, and recipient mice were sacrificed 4 weeks later. Representative flow cytometry plot of mature ILC2 in lungs and SILP of recipient mice with or without ILC2P transfer. (g) Number of ILC2 in the SILP or lung of recipient mice. Data are from 3 to 5 mice per group; *, *p* < 0.05; **, *p* < 0.01

We sought to understand why aging is paradoxically associated with increased early ILC2 development in the BM but reduced cellularity of mature ILC2 in the lung. We first examined the maturation capability of BM ILC2P in aged mice using established OP9 co‐culture assay (Hoyler et al., 2012). ILC2P sorted from the BM of aged mice differentiated into KLRG1^+^ mature ILC2 on OP9 stroma at a comparable efficiency as young ILC2P, indicating that the maturation capability of BM ILC2P does not decline with aging (Figure 3a,b). In addition, we detected both KLRG^‒^ immature ILC2P and KLRG1^+^ mature ILC2 in the peripheral blood of young and old mice, suggesting that active ILC2 maturation occurs in the circulation (Figure 3c). The numbers of both immature KLRG^‒^ ILC2P and mature KLRG1^+^ ILC2 were elevated with aging, which might reflect increased output from the BM (Figure 3c). The ratio of circulating KLRG1^+^ mature ILC2 versus KLRG1^‒^ immature ILC2P was comparable between young and old mice, verifying that the in vivo maturation of ILC2P is not impaired with aging (Figure 3d). Together, these data indicate that the ILC2P in aged mice do not exhibit maturation defects and that circulating mature ILC2 might be constantly replenished by immature precursors.

We further explored potential destination of aged BM ILC2P. Interestingly, in contrast to the reduced number of lung ILC2 in aged mice, the number of ILC2 in the small intestinal lamina propria (SILP) was greatly increased with aging (Figure 3e). To clarify the tissue homing potential of aged BM ILC2P, we sorted BM ILC2P from aged mice and transferred them intravenously to *Rag2^‒^* ^/^ *^‒^Il2rg^‒^* ^/^ *^‒^* recipient mice. At 4 weeks post‐transfer, we observed robust reconstitution of ILC2 in the small intestines, but not in the lungs of recipient mice (Figure 3f,g). Thus, aged BM ILC2P possess the capability to efficiently replenish mature ILC2 in the gut, but not those in the lungs. The lack of replenishment from BM precursors likely underpins aging‐induced decline in cellularity and function of lung ILC2.

### 2.4 Aged lung ILC2 display remarkable transcriptomic heterogeneity and have reduced expression of peroxisomal and cytochrome P450 enzyme genes that are required for optimal ILC2 function

**Figure 4:**
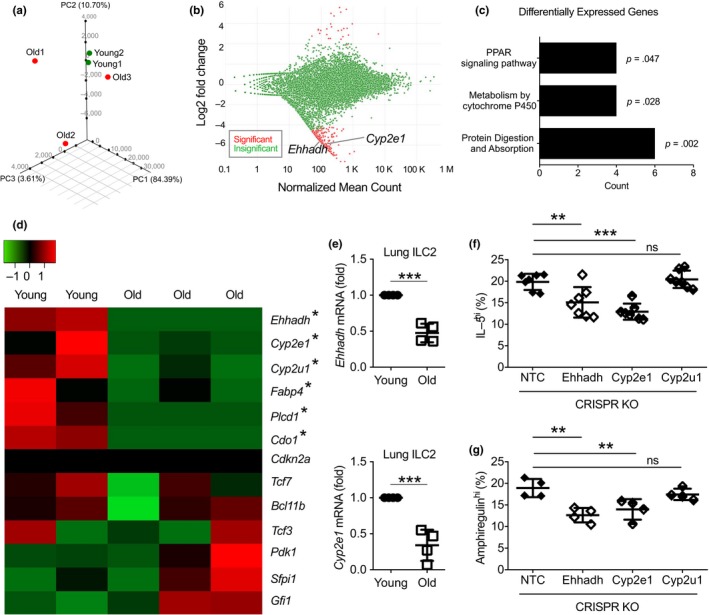
Aged lung ILC2 display genomic heterogeneity and have greatly reduced the expression of several metabolism genes required for optimal function of ILC2. (a) Principle component analysis of RNA sequencing data with lung ILC2 cells from young and old mice. (b) MA plot showing genes that are significantly altered (red) in young and aged lung ILC2. Y‐axis indicates fold change of gene expression in aged versus young ILC2. (c) Gene pathway analysis of differentially expressed genes in young and aged lung ILC2. (d) Heatmap depicting the expression of representative genes in aged versus young lung ILC2. (e) Expression of *Ehhadh* and *Cyp2e1* in young and aged lung ILC2 was verified by qPCR. Mature ILC2 were transduced with CRISPRv2GFP knockout lentivirus targeting *Ehhadh*, *Cyp2e1*, *Cyp2u1*, or nontarget control (NTC). (f) Expression of IL‐5 was examined by intracellular staining, and percentage of GFP^+^ ILC2 expressing high amounts of IL‐5 was quantified. (g) Expression of amphiregulin was examined by intracellular staining, and percentage of GFP^+^ ILC2 expressing high amounts of amphiregulin was quantified. Data are from 3 to 4 independent experiments; *, *p* < 0.05; **, *p* < 0.01; ***, *p* < 0.001

We next compared the transcriptomes of lung‐resident ILC2 in young and old mice by RNA sequencing. Principal component analysis (PCA) revealed remarkable heterogeneity in the transcriptomes of aged lung ILC2 (Figure 4a). These data are consistent with previous studies in other systems, suggesting that genomic heterogeneity is a hallmark of aging (Lopez‐Otin et al., 2013). Despite the great heterogeneity in the gene expression of aged lung ILC2, we still detected a small subset of genes whose expression levels were significantly altered with aging (Figure 4b–d). Interestingly, the genes downregulated in aged lung ILC2 were enriched for genes involved in peroxisome proliferator‐activated receptor (PPAR) pathway and cytochrome P450 (CYP) activity (Figure 4b–d). Young lung ILC2 expressed high amounts of peroxisomal and cytochrome p450 (CYP) enzyme genes, indicating high activities of peroxisome and cytochrome P450 in these cells (Figure 4d,e). Aged lung ILC2, however, had significantly reduced expression of multiple peroxisomal and CYP enzyme genes, including *Ehhadh, Cyp2e1*, and *Cyp2u1*, suggesting reduced peroxisome and CYP activity (Figure 4d,e). To determine whether these enzymes might affect the functional activity of ILC2, we deleted each individual enzyme in mature ILC2 using CRISPR‐mediated gene knockout technique. Deletion of *Ehhadh* and *Cyp2e1*, but not *Cyp2u1*, resulted in decreased production of IL‐5 and amphiregulin by mature ILC2 (Figure 4f, g). Deletion of either enzyme did not affect GATA3 expression in mature ILC2, indicating that *Ehhadh* and *Cyp2e1* might enhance ILC2 function through GATA3‐independent mechanisms (Figure S3). Our data thus reveal a novel role for *Cyp2e1* and *Ehhadh* in promoting ILC2 function. The reduced expression of these enzymes, as well as decreased expression of GATA3 protein (Figure 2c), likely together contributes to the reduced functional activity of lung ILC2 with aging.

Of note, treatment with anti‐Notch1 and anti‐Notch2 neutralizing antibodies did not affect the numbers of mature ILC2 in the lungs of old mice (Figure S4). Thus, Notch signaling specifically promotes aging‐associated accumulation of BM ILC2P, but does not affect the maintenance of mature ILC2 in the lungs of aged mice. In addition, the expressions of *p16* and *p16*‐interacting proteins were undetectable in either young or aged lung ILC2, suggesting that p16‐driven cellular senescence pathways might be repressed in lung ILC2 (Figure 4d). Aged lung ILC2 also had reduced expression of fatty acid transport gene *Fabp4*, which might explain the decreased fatty acid uptake by ILC2 with aging (Figure 4d). The expression of known transcriptional regulators of ILC2, such as *Tcf7*, *Bcl11b*, and *Gfi1*, was not reduced with aging (Figure 4d).

### 2.5 Increased levels of IL‐18 and IL‐12 in the aged lungs inhibit Cyp2e1 expression and repress mature ILC2 function

**Figure 5:**
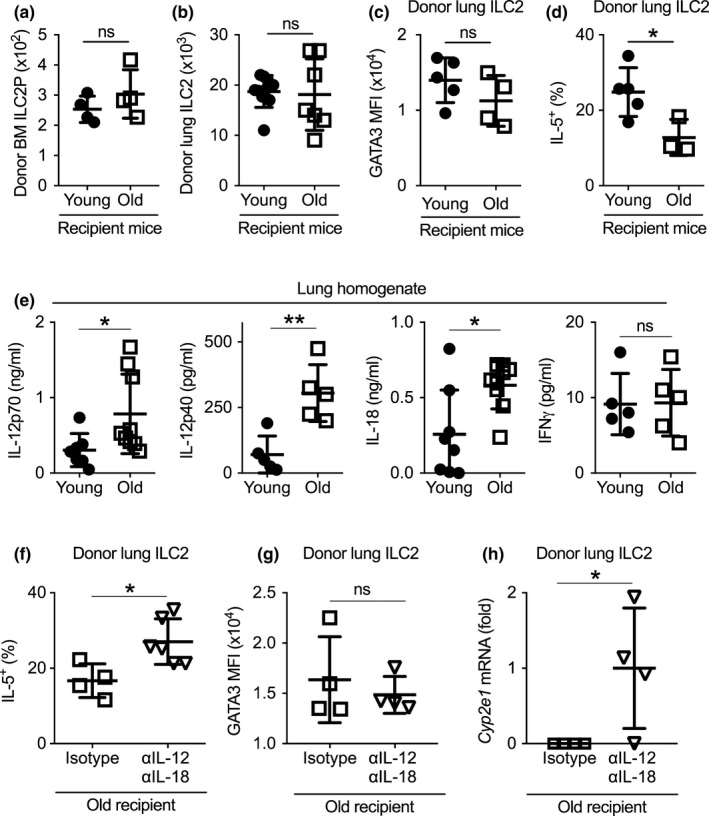
Increased levels of IL‐18 and IL‐12 in the aged lungs contributed to decreased *Cyp2e1* expression and reduced functional activity of mature lung ILC2. (a) Recipient young and old CD45.2 mice were irradiated and transplanted with bone marrow cells from young CD45.1 donor mice. Number of donor‐derived ILC2P in the bone marrow of recipient mice was examined at 4 weeks post‐transplant. (b) Number of donor‐derived mature ILC2 in the lungs of recipient mice was examined at 4 weeks post‐transplant (c) Mean fluorescence intensity (MFI) of GATA3 expression of donor‐derived lung ILC2 in young and old recipient mice. (d) Percentage of IL‐5^+^ donor ILC2 in the lungs of young and old recipient mice. (e) Cytokine concentrations from the lung homogenates of primary young and old mice. (f) Recipient young and old CD45.2 mice were irradiated and transplanted with bone marrow cells from young CD45.1 donor mice. Mice were treated with anti‐IL‐12p70 and anti‐IL‐18 antibodies or isotype control every other day. IL‐5 expression by donor‐derived lung ILC2 was examined at 4 weeks post‐transplant. (g) GATA3 expression by donor‐derived lung ILC2 in old recipient mice with treatment of anti‐IL‐12p70 and anti‐IL‐18 antibodies or isotype control. (h) *Cyp2e1* mRNA levels in donor‐derived lung ILC2 in old recipient mice with treatment of anti‐IL‐12p70 and anti‐IL‐18 antibodies or isotype control. Data are from 4 to 9 mice per group; *, *p* < 0.05; **, *p* < 0.01

Innate lymphoid cells are long‐lived cells, and thus, cell‐intrinsic mechanisms likely play a significant role in ILC aging (Lowery et al., 2013; Nussbaum et al., 2013). Nevertheless, aging also induces complex changes to the lung microenvironment (Lowery et al., 2013). We thus determined whether microenvironmental factors in the aged lungs might also contribute to ILC2 aging. We transplanted BM cells from young CD45.1 mice to irradiated young or old CD45.2 recipient mice. At 4 weeks post‐transplant, young donor BM cells efficiently repopulated BM ILC2P and gave rise to BM ILC2P and mature lung ILC2 in both young and old recipients after irradiation (Figure 5a,b). The numbers of donor‐derived ILC2P and mature ILC2 were comparable in young versus old recipient mice, indicating that aging‐related deregulation of ILC2 development and maintenance is likely due to hematopoietic cell‐intrinsic mechanisms or irradiation‐sensitive factors (Figure 5a). Donor‐derived mature lung ILC2 expressed comparable levels of GATA3 in young and old recipient, suggesting that the reduced GATA3 expression in aged lung ILC2 might also be due to cell‐intrinsic mechanisms (Figure 5c). However, donor young ILC2 still failed to efficiently produce IL‐5 in the lungs of aged recipient mice, indicating potential involvement of irradiation‐resistant microenvironmental factors (Figure 5d). IL‐33, TSLP, and IL‐25 are important activators of ILC2 (Artis & Spits, 2015), but their concentrations in the lungs were not reduced with aging (Figure S5). Using ELISA and multiplex assays, we noted aged lungs have increased concentrations of IL‐18 and IL‐12, two pro‐inflammatory cytokines that may regulate mature ILC2 activity (Figure 5e). The levels of IFNγ, another known regulator of ILC2, remained comparable between young and aged lungs at homeostasis (Figure 5e). Notably, inhibition of IL‐18 and IL‐12 by neutralizing antibodies enhanced IL‐5 production by donor‐derived lung ILC2 in old recipient mice, indicating that increased amounts of IL‐12 and IL‐18 contribute to decreased functional activity of mature lung ILC2 with aging (Figure 5f). Anti‐IL‐18 and anti‐IL‐12 treatment did not affect the expression of GATA3 (Figure 5g), but increased the expression of the cytochrome P450 oxidase *Cyp2e1* (Figure 5h). Because *Cyp2e1* is required for optimal ILC2 function (Figure 4f), increased IL‐18 and IL‐12 in the aged lungs might repress ILC2 function in part by inhibiting *Cyp2e1* expression. The expression of *Ehhadh* was undetectable in donor ILC2 of either group of recipient mouse (not shown). Together, our data indicate that aging leads to increased levels of IL‐12 and IL‐18 that suppress lung ILC2 function in part by inhibiting the expression of the cytochrome P450 oxidase *Cyp2e1*.

### 2.6 Transfer of activated young ILC2 enhanced resistance to influenza infection in old mice

**Figure 6:**
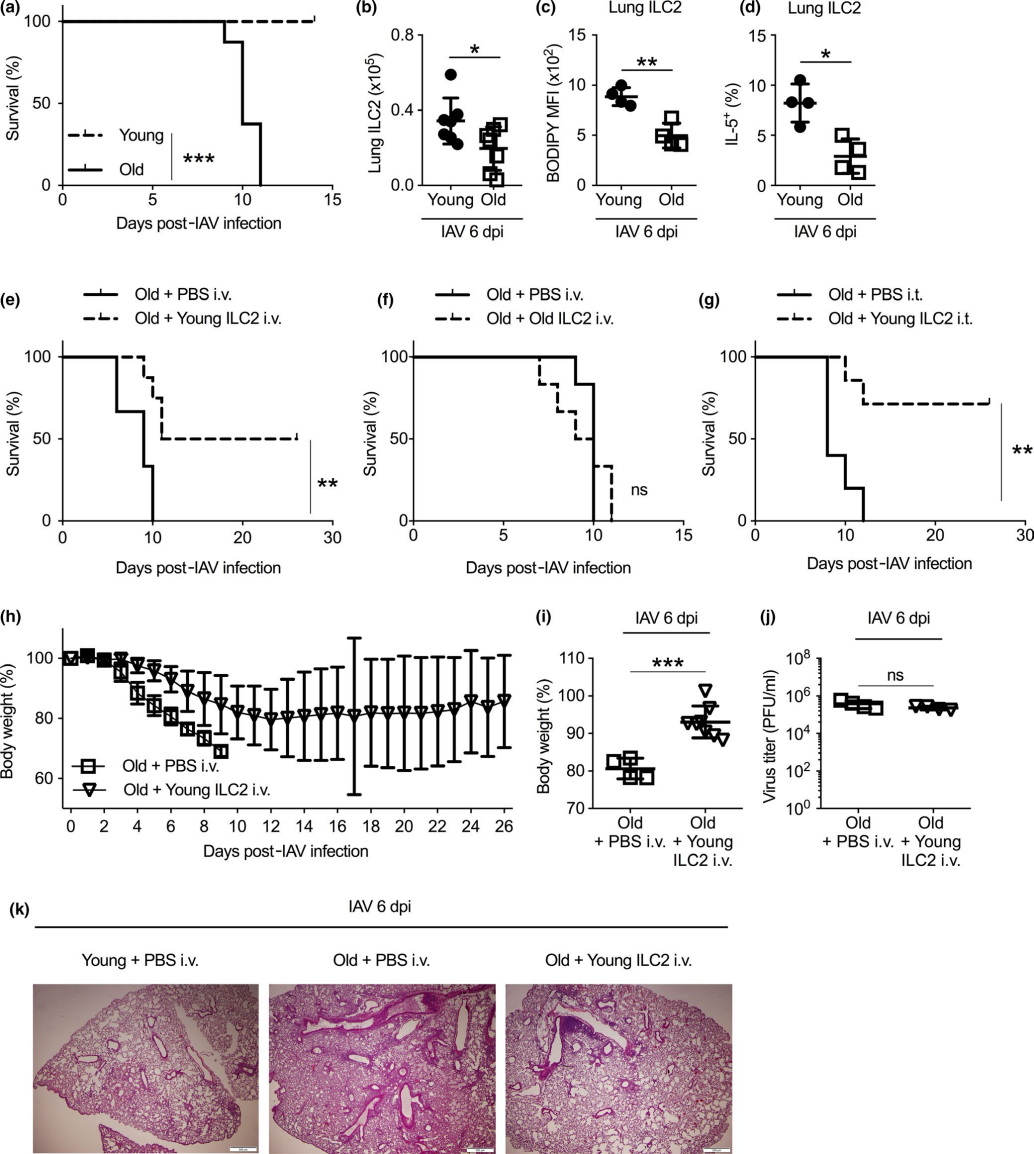
Adoptive transfer of young ILC2 enhanced resistance to influenza infection in old mice. (a) Survival rates were examined for young and old mice infected with 340 PFU of CA04 influenza A virus (IAV). (b) Number of lung ILC2 was examined at day 6 postinfection. (c) Uptake of BODIPY FL C_16_ by lung ILC2 was examined at day 6 postinfection. (d) IL‐5 expression by lung ILC2 was measured by flow cytometry at day 6 postinfection. (e) Old mice were transferred with sort‐purified lung ILC2 from young donor mice or administrated with PBS control via intravenous injection, and infected with 340 PFU of CA04 IAV. Survival was monitored up to 28 days postinfection. (f) Old mice were transferred with sort‐purified lung ILC2 from old donor mice or administrated with PBS control via intravenous injection, and infected with 340 PFU of CA04 IAV. Survival was monitored. (g) Old mice were transferred with sort‐purified lung ILC2 from young donor mice or administrated with PBS control via intratracheal injection, and infected with 340 PFU of CA04 IAV. Survival was monitored up to 28 days postinfection. (h) Weight loss of IAV‐infected old mice with or without intravenous transfer of young lung ILC2 cells. (i) Weight loss of IAV‐infected old mice at day 6 postinfection with or without intravenous transfer of young lung ILC2 cells. (i) Virus titer of IAV in infected old mice with or without intravenous transfer of young ILC2 cells. (k) Representative H&E staining of the lungs of infected young mice, infected old mice, or infected old mice received intravenous young ILC2 transfer, at day 6 post‐IAV infection. Data are from 8 mice per group (a), or 6 mice per group (e–g), or 4–6 mice per group (b–d, h, i); *, *p* < 0.05; **, *p* < 0.01; ***, *p* < 0.001

Influenza infection in the elderly remains a severe public health threat (Wilhelm, 2018). We thus examined the effects of aging on ILC2 responses during influenza infection. Infection with 340 PFU of influenza A H1N1 Cal/04/09 (H1N1 CA04) virus resulted in 100% lethality in old mice, while all young mice survived this dose (Figure 6a). Hence, old mice were more susceptible to influenza A virus (IAV) infection, mimicking human responses to influenza. The number of lung ILC2 in infected old mice was fewer than those in infected young mice, at 6 days after H1N1 CA04 infection (Figure 6b). Fatty acid uptake and IL‐5 production remained lower in aged lung ILC2 after influenza infection than those in young lung ILC2 (Figure 6c,d). Thus, lung ILC2 in aged mice remain numerically and functionally compromised during influenza infection.

We next determined whether transfer of young ILC2 into old mice might enhance their resistance to influenza infection. We obtained activated ILC2 from the lungs of IL‐33‐treated young mice and transferred 2 × 10^5^ cells into old mice intravenously. As we described before (Shen et al., 2018; Yang et al., 2013), IL‐33 treatment is necessary to expand lung‐resident ILC2 so that sufficient numbers of ILC2 can be obtained for adoptive transfer. Around 50% of old mice that received intravenous (*i.v*.) transfer of young lung ILC2 survived 340 PFU of CA04 infection, whereas all mice without young ILC2 transfer died (Figure 6e). In contrast, old mice transferred with the same number of aged lung ILC2 did not exhibit enhanced resistance to influenza infection, verifying that the beneficial function of lung ILC2 is lost with aging (Figure 6f). Increased survival was also observed when young ILC2 were delivered directly into the airway of aged mice via intratracheal (*i.t*.) transfer (Figure 6g). Weight loss was greatly improved with *i.v*. transfer of young ILC2 (Figure 6h,i). Viral burdens, however, remained similar in the lungs of old mice with or without young ILC2 transfer, indicating that the protective function of ILC2 might be independent of viral clearance (Figure 6j). Lung inflammation was markedly alleviated in mice that received young ILC2 transfer, suggesting that the improved survival might be associated with reduced inflammation (Figure 6k). Together, transfer of activated young lung ILC2 enhanced resistance to influenza in old mice.

## 3 DISCUSSION

The present work reveals compartmentalized effects of aging on ILC2 development and function, and suggests that replenishing tissue‐resident ILC2 might provide new avenues to enhance resistance to infections and diseases in the aged population. Although early ILC2 development and maturation remain active in aged mice, the newly generated ILC2 might not settle in the lungs to replenish tissue‐resident mature ILC2. With the lack of replenishment of new cells, mature ILC2 in the aged lungs were numerically and functionally compromised. They displayed great transcriptomic heterogeneity and failed to produce cytokines due to multiple cell‐intrinsic mechanisms and microenvironmental factors. Transfer of young lung ILC2 enhanced resistance to influenza in old mice. Together, this work reveals a novel mechanism of immunosenescence and highlights complex tissue‐specific mechanisms of innate lymphoid cell aging.

Despite the lack of adaptive antigen receptors, ILCs are classified into the lymphocyte family based on their morphological, molecular, and developmental similarity with adaptive T/B lymphocytes (Vivier et al., 2018; Yang, Saenz, Zlotoff, Artis, & Bhandoola, 2011). However, although adaptive T/B lymphopoiesis is diminished with aging (Chinn et al., 2012; Goronzy & Weyand, 2013; Nikolich‐Zugich, 2018), early ILC2 development in the BM is instead drastically increased in old mice due to enhanced Notch signaling. Our results thus challenge the conventional view about a global decline in lymphopoiesis with aging and indicate that aging is associated with diametrically opposed effects on innate versus adaptive lymphopoiesis. Since early hematopoiesis is likely a gradual process mediated by a variety of known and unknown precursors with overlapping but distinct developmental potentials and lineage biases (Dorshkind, 2010), it is possible that subsets of ILC‐primed lymphoid precursors with enhanced susceptibility to Notch signaling are relatively spared by the effects of aging‐related BM stromal cell failure. The unique molecular or epigenetic features of these precursors might lead to a strong bias toward the ILC2 lineages during later developmental stages. Nevertheless, the newly generated ILC2 and immature precursors might not be able to settle in the lungs to replenish the concomitantly declining mature lung ILC2 pool, resulting in futile developmental efforts. Thus, strategies to enhance precursor homing into the lungs might hold promises to enhance resistance to infections and diseases in the aged population, by allowing replenishment of newly generated “young” ILC2 in the aged lungs.

Not only do aging‐related changes in early ILC2 development differ from those in adaptive lymphocyte development, but aged mature lung ILC2 also exhibit many unique characteristics that differ from the aging of other cell types. Aging or senescence has been associated with enlarged cell size, enhanced lipid peroxidation, and senescence‐associated secretory phenotype in other cell types (Biran et al., 2017; Coppe, Desprez, Krtolica, & Campisi, 2010; Flor, Wolfgeher, Wu, & Kron, 2017; Petursdottir, Farr, Morley, Banks, & Skuladottir, 2007). However, these phenotypes have not been observed in aged lung ILC2. In contrast, aged lung ILC2 are smaller in size and have reduced cytokine secretion capability. In addition, aged lung ILC2 have reduced fatty acid uptake and decreased expression of peroxisomal and cytochrome p450 enzymes. Finally, the expression of p16 is not upregulated in aged lung ILC2, suggesting that cellular senescence pathways are not activated with aging in mature lung ILC2. Because lung ILC2 are long‐lived slow‐cycling cells, it is possible that p16‐driven cellular senescence pathways have been repressed in these cells and that other aging‐related mechanisms might play predominant roles. Together, our data indicate that unique cellular and molecular pathways underlie the aging of long‐lived tissue‐resident innate lymphocytes, likely due to the combined effects of multiple cell‐intrinsic mechanisms and microenvironmental factors.

Our data also identify a novel role for the cytochrome oxidase *Cyp2e1* in promoting lung ILC2 function. *Cyp2e1* is a major liver cytochrome P450 oxidase involved in drug metabolism and ω‐1 hydroxylation of fatty acid (Porubsky, Meneely, & Scott, 2008). The Th2 cytokine IL‐4 can induce the expression of *Cyp2e1* in hepatocytes during acute liver injury (Abdel‐Razzak et al., 1993). *Cyp2e1* is also expressed in lymphocytes of human diabetes patients (Hannon‐Fletcher, O'Kane, Moles, Barnett, & Barnett, 2001). *Cyp2e1* may promote the proliferation of CD4 T‐cell proliferation and the activity of CD8^+^ CD57^+^ cytotoxic T cells (Griffin, Gilbert, & Pumford, 2000; Seth et al., 2014). But the precise role of *Cyp2e1* in lymphocyte function remains unknown. Interestingly, our RNA‐seq data indicate that lung ILC2 in young mice express high amounts of *Cyp2e1* and other cytochrome P450 genes, indicating high activity of cytochrome P450. CRISPR‐mediated gene knockout technique revealed that *Cyp2e1* is required for optimal ILC2 function. More importantly, aging increases the levels of pro‐inflammatory cytokines IL‐12 and IL‐18 that repress *Cyp2e1* expression in tissue‐resident ILC2, leading to reduced ILC2 function. Together, these data indicate that altered cytochrome P450 activity is associated with the aging of tissue‐resident innate lymphocytes.

Respiratory and other infections remain severe threats in the aged population. More than 70% of influenza‐related deaths occur among people over 65 years old (Wilhelm, 2018). ILC2, the predominant ILC subset in the lung, have been implicated in host defense against influenza infection in several previous studies (Califano et al., 2018; Gorski, Hahn, & Braciale, 2013; Li et al., 2018; Monticelli et al., 2011). But the precise role of ILC2 in pulmonary homeostasis and airway infections remains to be delineated. In this study, we have provided direct evidence that transfer of activated young ILC2 can enhance resistance to influenza infection in old mice. The protective function of ILC2 is independent of viral clearance, but is likely associated with alleviated airway inflammation and injury. Of note, aging is also associated with well‐documented changes in other immune cell types. In particular, naive T and B cells are diminished with aged mice and humans, whereas memory and memory‐like T and B lymphocytes with dysregulated function accumulate with aging (Fukushima, Minato, & Hattori, 2018; Hao, O'Neill, Naradikian, Scholz, & Cancro, 2011). Whether and how the aging‐associated changes in adaptive immunity may influence ILC2 activity warrants future investigation. Understanding mechanisms of innate lymphoid cell aging might suggest new opportunities to enhance resistance to infections and diseases in the elderly.

## 4 EXPERIMENTAL PROCEDURES

### 4.1 Mice

Young (2–3 month) and old (19–24 months) female C57BL/6 mice were obtained from National Institute of Aging via Charles River, or purchased from Charles River, or bred in the animal research facility of Albany Medical Center. B6‐LY45.2 (CD45.1) mice were purchased from Charles River. *Rag2* ^‒/‒^ *Il2rg* ^‒/‒^ mice were purchased from Taconic (Model #4111). All animal experiments were performed according to protocols approved by the Institutional Animal Care and Use Committee at Albany Medical Center.

### 4.2 Bone marrow transplantation and antibody treatment

For bone marrow transplantation, donor BM cells from young CD45.1 mice were depleted of Thy1.2^+^ T cells and ILCs using Thy1.2 magnetic beads (Miltenyi). 3 × 10^6^ cells were *i.v*. transferred to irradiated (800rads) young and old C57BL/6 mice recipient mice (CD45.2). Donor‐derived cells were examined at 4 weeks post‐transplant. For IL‐18‐ and IL‐12‐neutralizing antibody treatment, recipient mice were administrated intraperitoneally (*i.p*.) with 600 μg IL‐18 and 600 μg IL‐12 mAbs (Bio X Cell) every other day starting from the day of bone marrow transplant.

For anti‐NRR1 and anti‐NRR2 antibody treatment, neutralizing mAbs specific for Notch1 or Notch2 (Wu et al., 2010) were injected i.p. at 5 mg/kg on days 0 and 3. Mice were euthanized at day 4.

### 4.3 ILC2P adoptive transfer

For ILC2P adoptive transfer, 10,000 ILC2P were sorted from bone marrow of old mice and injected intravenously into nonirradiated *Rag2* ^‒/‒^ *Il2rg* ^‒/‒^ mice. ILC2 reconstitution in lung and SILP was examined at 4 weeks post‐transfer.

### 4.4 Influenza Infection, antibody treatment, and adoptive transfer

For influenza infection, 340 PFU of influenza A virus CA04 strain was administered to mice via the intranasal route. Mice survival and body weight were monitored daily up to 28 days postinfection. ILC2 responses were examined at day 6 postinfection. Viral titers were measured using a standard viral plaque assay on MDCK cells.

For adoptive ILC2 transfer, ILC2 were sorted from young mice receiving 300 ng of recombinant mouse IL‐33 (BioLegend) for 7 days. 2 × 10^5^ cells were transferred i.v. or i.t. into each old mouse the day before influenza infection and again at 6 days postinfection.

### 4.5 Isolation of hematopoietic cells from the lungs and small intestinal lamina propria

Lungs were harvested after perfusion with 10 ml of cold PBS. Tissues were minced using scissors and digested in Hank's balanced salt solution containing 0.2 mg/ml Liberase TM (Roche) and 0.1 mg/ml DNase I (Roche) for 30 min at 37°C. Single‐cell suspension was prepared by passing the tissue through an 70‐μM cell strainer. SILP lymphocytes were isolated as we described previously (Spencer et al., 2014; Yang et al., 2015). Briefly, tissues were incubated with an epithelial strip buffer (alpha‐MEM medium with 1 mM EDTA, 1 mM DTT, and 5% FCS) for 30 min at 37°C, followed by digestion with 10 ml liberase TM solution for another 20 min. Lymphocytes were further purified using 40% Percoll gradient centrifugation.

### 4.6 Flow cytometry and cell sorting

Antibodies (Abs) were purchased from BioLegend or eBioscience or MD Bioproducts. Abs in the lineage cocktail included anti‐CD3 (2C11), anti‐B200 (RA3‐6B2), anti‐Mac‐1 (M1/70) (8C5), anti‐NK1.1 (PK 136), anti‐TCRβ (H57), and anti‐γδTCR (GL‐3). Other Abs used included anti‐CD45.2 (104), anti‐CD45.1 (A20), anti‐Sca‐1 (D7), anti‐CD25 (PC61.5), anti‐Thy1.2 (53‐2.1), anti‐CD127 (A7R34), anti‐α4β7 (DATK32), anti‐IL‐5 (TRFK5), anti‐amphiregulin (R&D Systems BAF989), anti‐ST2 (DJ8), anti‐GATA3 (TWAJ), anti‐PLZF (Mags.21F7), anti‐Rorγτ (B2D), and anti‐T‐bet (4B10). Intracellular staining of transcription factors was performed using Foxp3 Fix/Perm Kit (Thermo) according to the manufacturer's instructions. Flow cytometric analysis was performed on FACSCanto (BD Bioscience) or LSRII (BD Bioscience). Cell sorting was performed using an FACSAria II (BD Biosciences).

### 4.7 CRISPR‐mediated gene knockout

LentiCRISPRv2GFP was a gift from David Feldser (Addgene plasmid # 82416). Guide RNA (gRNA)‐encoding sequences were cloned into LentiCRISPRv2GFP vectors to achieve gene knockout effects as described (Sanjana, Shalem, & Zhang, 2014). The following gRNA sequences were used: AGCTGCGTTCCTCTTGCACC (targeting *Ehaddh*), CCACATGGAAGGACGTGCGG (targeting *Cyp2e1*), CACTCGACGCTTCGTCATTT (targeting *Cyp2u1*), and TGCGAATACGCCCACGCGATGGG (nontarget control). Lentiviral supernatant was prepared with 293T cells by co‐infection of the LentiCRISPRv2GFP vector and the packaging vectors psPAX2 and pM2.G. Mature ILC2 were cultured for 7 days with 10 ng/ml of IL‐2, IL‐7, and IL‐33, and transduced with the indicated CRISPRv2GFP lentivirus by spin infection at 1,000 g for 1.5 hr as we previously described (Yang et al., 2013). Cytokine production was examined by intracellular staining after 7 days post‐transduction.

### 4.8 BODIPY and 2‐NBDG treatment

Mice were injected *i.p*. with 50μg of BODIPY FL C16 (Thermo) or 100 µg 2‐NBDG (Cayman Chemicals). Mice were euthanized at 60 min after BODIPY treatment or 20 min after 2‐NBDG treatment. Cells were isolated and stained with surface markers followed by flow cytometric analysis for examination of BODIPY and 2‐NBDG uptake.

### 4.9 Cytokine measurement

Cytokine concentrations of lung homogenate were measured by LEGENDplex (BioLegend) or ELISA (R&D) kits according to the manufacturers' instructions. For intracellular cytokine staining in ILC2, cells were incubated with monensin for 2.5 hr at 37°C. Intracellular cytokine staining was performed using the Fix/Perm Kit (BD Biosciences).

### 4.10 RNA sequencing

For RNA sequencing, each sample was pooled with sorted ILC2 from two mice. Cells were sorted into TRIzol LS. Total RNA was extracted, and amplified cDNA was generated using the SMART‐Seq v4 Ultra Low Input RNA Kit (Takara). Libraries were generated with Nextera XT DNA Library Prep Kit (Illumina). Single‐end 75 bp high‐throughput sequencing was performed using a NextSeq 500 (Illumina). Raw data were converted to fastq files using bcl2fastq (v2.20.0.422) and quality‐checked using fastqc (v1.0.0). Data were aligned and normalized with the STAR aligner (v2.5.3a) followed by differential expression with DESeq2 (v1.1) (Dobin et al., 2013; Love, Huber, & Anders, 2014). Gene pathway analysis was performed with DAVID (da Huang, Sherman, & Lempicki, 2009a, 2009b). RNA‐seq data were deposited at GEO (accession number: GSE125584).

### 4.11 Reverse transcription‐quantitative PCR (RT–qPCR)

For RT–qPCR analysis, mRNA was purified from sorted young and old lung ILC2 using TRIzol LS (Thermo) or RNeasy Plus Mini Kit (Qiagen) according to manufacturer's protocol. cDNA was generated using SuperScript II Reverse Transcriptase (Thermo). qPCR was performed on a StepOnePlus RT‐PCR System (Applied Biosystems) using ABI TaqMan probes to detect *Ehhadh (*Mm00619685_m1*)*, *Cyp2e1* (Mm00491127_m1), and *Gapdh* (Mm99999915_g1). Relative gene expression was calculated and normalized to *Gapdh*.

### 4.12 Statistics

Difference between two groups was examined by two‐tailed Student's *t* tests. Difference between more than two groups was examined by one‐way ANOVA. Survival rates between groups were assessed by log‐rank (Mantel–Cox) test. *p* < 0.05 was considered significant.

## CONFLICT OF INTERESTS

The authors declare no competing financial interest.

## AUTHOR CONTRIBUTIONS

S. D'Souza. and Q. Yang designed the study and wrote the manuscript. S. D'Souza. performed most of the experiments. X. Shen, I. Fung. L. Y, and Q. Yang performed some experiments. M. Kuentzel and S.V. Chittur performed the RNA sequencing experiments. Y. Furuya, D.W. Metzger, C. W. Siebel, and I. P. Maillard provided essential research tools, helped design and interpret some experiments, and edited the manuscript. All authors reviewed and approved the manuscript.
