# Supplementary figures and images for: Compartmentalized effects of aging on group 2 innate lymphoid cell development and function
Source: Aging Cell. 2019 Aug 20;18(6):e13019. doi: 10.1111/acel.13019 (PMC6826140; doi:10.1111/acel.13019)

Figure S1

A

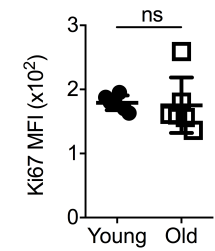

B

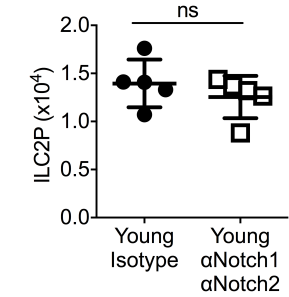

Supplement: Supplementary file 1 [file ACEL-18-e13019-s001.pdf]

Figure S2

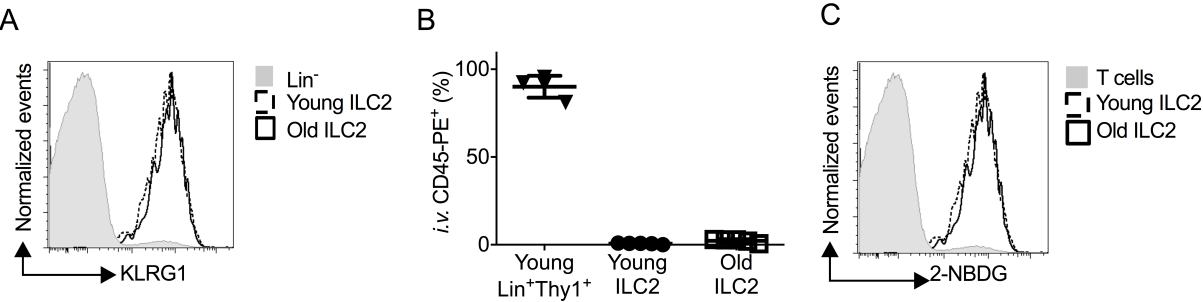

Supplement: Supplementary file 2 [file ACEL-18-e13019-s002.pdf]

### Figure S3

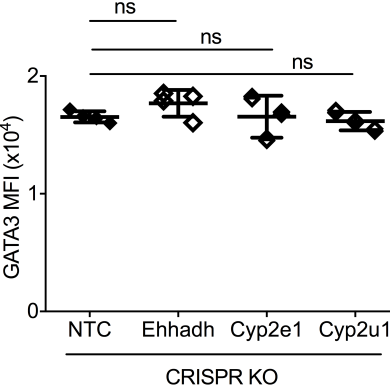

Supplement: Supplementary file 3 [file ACEL-18-e13019-s003.pdf]

Figure S4

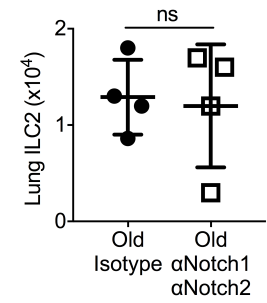

Supplement: Supplementary file 4 [file ACEL-18-e13019-s004.pdf]

Figure S5

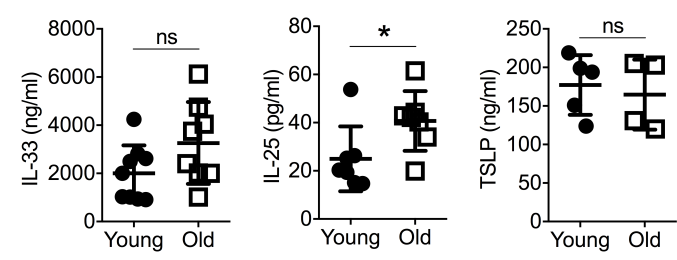

Supplement: Supplementary file 5 [file ACEL-18-e13019-s005.pdf]
